# Geographic Variation of Song Sharing in Birds—A Meta‐Analysis

**DOI:** 10.1002/ece3.74566

**Published:** 2026-10-20

**Authors:** Mónika Jablonszky, László Zsolt Garamszegi

**Affiliations:** ^1^ Evolutionary Ecology Research Group, Institute of Ecology and Botany HUN‐REN Centre for Ecological Research Vácrátót Hungary; ^2^ Behavioural Ecology Group, Department of Systematic Zoology and Ecology ELTE Eötvös Loránd University Budapest Hungary

**Keywords:** acoustic communication, Aves, bird song dialects, MCMCglmm, phylogenetic meta‐analysis, urbanization

## Abstract

Bird song plays a crucial role in sexual selection and often exhibits geographical variation, with neighboring birds typically singing more similar songs. Song sharing, created by birds learning songs or song elements from their neighbors or matching each other's song, is a key focus of research in this aspect. In this meta‐analysis, we quantified the overall effect of geographic distance on song sharing and investigated the influence of migratory tendency, repertoire size, dichromatism (as a proxy for sexual selection intensity), and habitat (urban, rural, and natural) on the focal relationship. We confirmed that song sharing decreases with geographic distance but found no overall effect of migration or repertoire size. Interestingly, this association was stronger negatively in monochromatic species and suburban habitats, potentially indicating that dichromatic species prioritize individuality in their vocal displays, while suburban birds may benefit from increased song sharing to enhance signal transmission or reduce competition. Our findings emphasize the importance of considering multiple factors when studying geographical variation in bird song. Future research should investigate other measures of song similarity and the roles of social structure and learning mechanisms.

## Graphical abstract

Using a phylogenetic meta‐analytic framework, we investigated whether closer birds share more songs in general and what species‐level factors (migratory tendency, song complexity, sexual dichromatism, and habitat) influence this pattern. We found a general pattern of more shared songs in closer birds that was stronger in monochromatic birds and in suburban habitats, suggesting that dichromatic species (where sexual selection is expected to be higher) share fewer songs with their neighbors to advertise their individuality for potential mates, and more urbanized areas may necessitate more song sharing to produce vocalizations spreading efficiently under these conditions or due to increased competition.

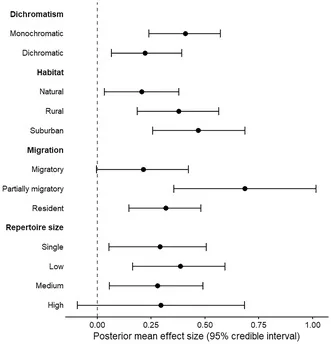

## 1 Introduction

The song of birds plays an important role in communication as an honest signal of sexual selection (Gil and Gahr 2002; Catchpole and Slater 2008). The within‐species variation of vocalizations (which can be very high) often shows a spatial pattern first described for the song of passerines (Marler and Tamura 1962). Studying this variation is important because it allows us to infer learning and movement processes (Slater 1989; Podos and Warren 2007; Ranjard et al. 2017) and because the spatial divergence in song, an important mating signal, can ultimately lead to speciation (Marler and Tamura 1962; Irwin et al. 2001; Slabbekoorn and Smith 2002). Spatial variation in song manifests as dialects or microdialects with clear boundaries (Marler and Tamura 1962; Leader et al. 2000; MacDougall‐Shackleton and MacDougall‐Shackleton 2001) or clinal variation (Morton 1987; Förchler and Kalko 2007; Lee et al. 2019). The most common pattern, which has been observed across various spatial scales, is that closer birds sing more similarly (Sung and Handford 2006; Quiroz‐Oliva and Sosa‐López 2022; Maldonado‐Coelho et al. 2023). In contrast, neighbors of certain species may actually have more dissimilar songs (Laiolo 2011; Garamszegi et al. 2012) and many studies failed to find a clear spatial pattern in vocalization similarity (Park et al. 2009; Riegert and Juzlová 2018). Despite widespread interest, the underlying mechanisms driving this variation remain poorly understood, and few empirical studies have investigated multiple potential causal factors simultaneously.

Song similarity describes the degree of resemblance between songs produced by different individuals and can be quantified using a range of acoustic or structural measures. Importantly, song similarity can be assessed either through continuous measures of acoustic or temporal resemblance, for example, based on singing rates (Snijders et al. 2015), frequencies (Galeotti et al. 1997; Foote et al. 2008; Budka and Osiejuk 2017), or through discrete measures such as song sharing quantifying the occurrence of the same song types or song elements in the repertoires of different individuals (Camacho‐Schlenker et al. 2011; Sethi et al. 2014; Barišić et al. 2018). Thus, although song sharing can contribute to acoustic similarity, these measures capture different aspects of vocal resemblance and may be shaped by partially distinct processes. Here, we focused on song and song element sharing that can arise through cultural transmission and social learning and has been widely investigated (Slater 1989; Gaunt et al. 1994; Fayet et al. 2014). Although song sharing is most directly relevant to interactions among neighboring or socially connected birds, spatial patterns in song sharing can extend over large geographic scales (Pavlova et al. 2012; González and Ornelas 2014; Pitocchelli et al. 2018). Song sharing is commonly interpreted as a consequence of cultural transmission because song types and elements can be acquired through social learning (Beecher et al. 1994; Johnson 2006; Ortega et al. 2014). However, the occurrence of a shared song type between two individuals does not necessarily demonstrate direct cultural transmission, as both may have acquired the same song type from a common tutor or from a shared population‐level song tradition.

Several non‐mutually exclusive hypotheses have been proposed regarding the factors creating and maintaining spatial patterns in vocalization similarity, including sexual selection, signaling group membership or familiarity, adaptation to habitat and cultural or genetic drift (Wright and Dahlin 2018). The sexual selection hypothesis proposes that singing similar songs within a locality may provide birds with advantages during both mate choice and intrasexual (most commonly male–male) competition, making song sharing advantageous. Females of various bird species were repeatedly found to prefer similar songs to their mate's or local dialects (Baker, Bjerke, et al. 1987; Mortega et al. 2014), probably because this facilitates mate recognition, species recognition (Haavie et al. 2004; Hamao et al. 2016; Nwankwo et al. 2018) or because this may indicate greater familiarity with local resources or territory quality (Lampe and Slagsvold 1998; O'Loghlen and Beecher 1999; Podos and Warren 2007). Song sharing also seems to lower aggression from neighbors, as it was associated with territory tenure (Beecher et al. 2000, 2020; Wilson et al. 2000) and less aggressive territorial response (Lovell and Lein 2004; Briefer et al. 2008; Thomas et al. 2021). So, we can expect stronger local song sharing and consequently stronger decline in sharing with geographic distance under stronger sexual selection. Conversely, low song sharing in birds singing near to each other can also be explained by social processes. Nearby birds may sing differently to make themselves more easily distinguishable or recognizable (Vargas‐Castro 2015; Hamao et al. 2016).

Effects from the habitat can shape songs (Morton 1975; Wiley and Richards 1982; Garcia et al. 2023) and also the patterns of song sharing (van Dongen and Mulder 2006; Schook et al. 2008), as one advantage of song learning can be specifically the ability to adjust vocalization to the prevailing acoustic conditions (Slater 1989). A relevant habitat‐related factor worth studying is urbanization, especially considering its continuous expansion. Urban environments can have diverse effect on bird song (Slabbekoorn and Ripmeester 2008) by altering the physical structures and consequently changing the transmission conditions (Slabbekoorn and den Boer‐Visser 2006; Phillips et al. 2020), fragmenting the suitable habitats and affecting population density (Laiolo 2011; Narango and Rodewald 2015). Thus we can expect differences in geographic variation in song sharing between urban and rural/natural habitats, but there are two competing predictions. On one hand, a weaker pattern should emerge in urban areas due to reduced song learning if birds have fewer interactions there because the increased isolation (Laiolo 2011) or due to the masking effect of noise (Rheindt 2003; Slabbekoorn and Ripmeester 2008; Fossesca et al. 2023). On the other hand, urbanization can also lead to higher population density and/or competition in suitable habitat patches (e.g., due to fragmentation by built‐up surfaces or more stable climate and high resource availability throughout the year (McKinney 2006; Shochat et al. 2006; Patankar et al. 2021)), and higher density or competition can result in high song sharing in neighboring and less sharing in more distant birds (Laiolo and Tella 2005; Petrusková et al. 2010). This could produce a stronger negative association between song sharing and geographic distance in cities.

Geographical pattern in song sharing could also arise due to cultural drift. If song traditions diverge through cultural drift, divergence is expected to accumulate with geographic distance, resulting in lower song sharing between more distant individuals. If this process contributes to spatial variation in song sharing, we therefore expected a stronger negative association between song sharing and geographic distance at larger spatial scales. (Lovell and Lein 2013; Schoen et al. 2023).

Empirical support is scarce for these hypotheses. One review on parrot calls suggested that local call similarity emerges due to a social advantage of similar calls; however, the authors warned about the lack of tests of other mechanisms (Wright and Dahlin 2018). Similarly, a review of geographic variation in bird song emphasized the need to study the mechanisms of this phenomenon (Sebastián‐González and Pérez‐Granados 2025). Another review of song dialects stressed that probably more mechanisms lead to the geographical patterns and that evidence supports indirect mechanisms (through other functions of song learning or morphology) and not adaptive ones (Podos and Warren 2007).

Regardless of the causing process involved, repertoire size and mobility may play important roles in determining spatial patterns of vocal variation (Handley and Nelson 2005; Podos and Warren 2007). In many species, birds from the same locality share similar songs (Beecher et al. 1994; Nelson and Poesel 2009; Thomas et al. 2021); however, several species show relatively little song sharing among neighbors (Wiley 2005; Soha et al. 2009; Palmero et al. 2012), and this pattern has been associated with longer migration distances (Kroodsma et al. 1999; Nelson et al. 2001; Yoon et al. 2013). High dispersal and migration could weaken the persistence of local song traditions if individuals experience greater turnover or variation in their social and learning environments across years. Migratory individuals may encounter different tutors or social environments after returning to their breeding grounds, potentially reducing the consistency with which local song traditions are maintained. We therefore predicted that migratory status would influence the strength of the negative association between song sharing and geographic distance, with more mobile species expected to show weaker geographic structuring of song sharing. However, this effect should depend strongly on the timing and type of song learning (Ellers and Slabbekoorn 2003; Beecher and Brenowitz 2005) that is unfortunately known only for a few species. For example, species that continue to learn or modify songs after arriving at their breeding grounds may still readily acquire songs from local neighbors, whereas species in which song crystallization occurs before migration may be less responsive to the local song environment.

The potential effect of repertoire size on the geographic patterns of song sharing is not straightforward. Large repertoires have been hypothesized to reduce song sharing because song‐learning strategies may not be able to optimize both repertoire size and the acquisition of songs shared with social partners (Williams and Slater 1990; Beecher et al. 2000; Beecher and Brenowitz 2005). Thus, species with larger repertoires may show less pronounced geographic variation in song sharing and, consequently, a weaker negative association between song sharing and geographic distance. However, a phylogenetic analysis in Fringillidae found no relationship between repertoire size and song sharing (Handley and Nelson 2005).

In this meta‐analysis, we aimed to estimate the overall strength of association between geographic distance and song sharing and to comprehensively explore the support for proposed mechanisms and potential influencing factors. Effect sizes represented the correlation between geographic distance and song sharing. Thus, a more negative effect size indicated a stronger negative association, whereby song sharing decreased more strongly with increasing geographic distance. We considered the effect of repertoire size and migratory status on this relationship and used dichromatism, habitat, and the geographical scale of the studies to differentiate between the potential mechanisms. We predicted that the effect of drift (less song sharing between birds farther apart) will appear more clearly if we examine it over larger distances. Sexual dichromatism has been used as an indirect measure of the intensity of sexual selection (Andersson 1994; Jeschke and Kokko 2008; Seddon et al. 2013). We therefore used dichromatism as a proxy for sexual selection and predicted that the strength of the relationship between song sharing and geographic distance would vary with dichromatism. If strong sexual selection favors conformity to local tradition, we expected a stronger negative relationship between song sharing and geographic distance in dichromatic species. We predicted that the negative association between song sharing and geographic distance would be weaker in species with larger repertoires and in migratory birds because lower overall song sharing should result in less pronounced geographic variation in song sharing in these groups. We expected that urbanization will affect the strength of association among species, but we did not state specific predictions due to the opposing hypothesis in the literature.

## 2 Methods

### 2.1 Data Collection

We systematically searched the Web of Science Core Collection Database in 2024 for studies testing the relationship between geographic distance and song similarity. We used the combinations of the following keywords: bird*, song*, distance, proximity, neighbor and *geograph*. We limited the search to English‐language publications. We also screened the reference lists of relevant reviews and primary articles to identify additional studies.

We included studies that quantitatively assessed the relationship between geographic distance and song similarity, provided data suitable for calculating correlation effect sizes, and focused on within‐species vocal variation. We excluded studies that were conducted on captive or translocated birds, reported only differences between groups of birds in some song characteristics (without accounting for geographic distance), and investigated the change in song traits (without quantifying their similarity) along a gradient. We also excluded studies that investigated song similarity between species or in females (as the sample size was too low for performing formal analysis in this sex).

Given the substantial heterogeneity of the variables used to calculate the focal relationship, we had to apply further eligibility criteria. Regarding song similarity, we initially retained all studies with variables describing some kind of similarity between the vocalization of two birds (or populations), such as sharing song types or elements of songs, spectro‐temporal similarity, dialect categories. However, as many of these categories had low sample sizes, we chose to focus on studies that quantified the association of song or song element sharing with distance, which category had substantially larger sample sizes and strong relevance to understanding cultural and learning processes (Beecher et al. 1994; Koetz et al. 2007; Nelson and Poesel 2009). Regarding distance, we included all studies that approximated the direct line geographic distance between two birds, including distance measured as the number of territories between the birds, transformed geographic distance, but excluding latitude and longitude. The PRISMA chart of the searching and screening processes can be seen on Figure 1. A list of data sources used in the study is provided in the Data sources section.

**FIGURE 1:**
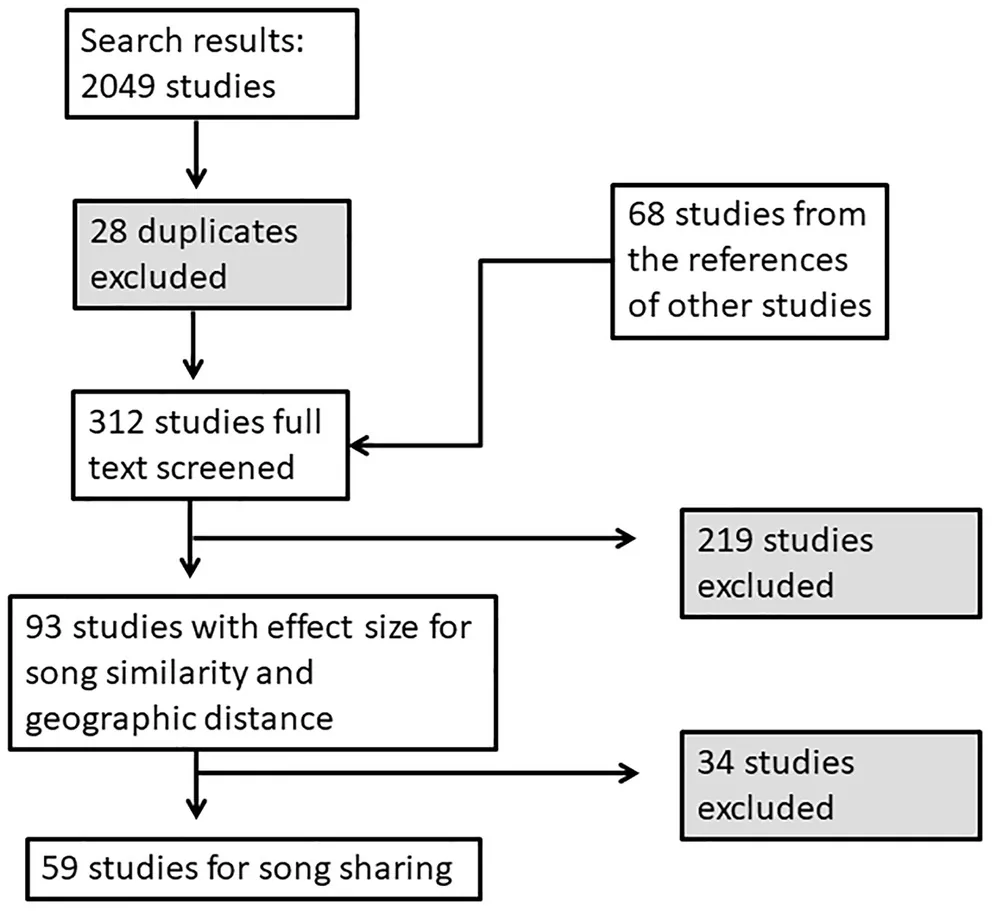
PRISMA chart of the search and screening processes, showing the number of studies identified, screened, and included in the meta‐analysis.

Furthermore, we classified data into independent sampling groups based on recording period and location and excluded studies with overlapping data. Effect sizes based on birds recorded at the same site 1–2 years apart were treated as belonging to one sampling group, whereas the two cases in which recordings were separated by ≥ 6 years were treated as two independent sampling groups (no cases with intervals of 3–5 years were available). The 6‐year interval represented a substantial period relative to the mean lifespan of the focal species ( *Chersophilus duponti* and *Emberiza schoeniclus* ), which was approximately 3 years (García‐Antón and Traba 2021; British Trust for Ornithology 2026), and was therefore expected to result in substantial turnover of individuals.

In addition to the focal relationship, we extracted the following information from the studies: maximum distance between the recorded birds (to characterize the spatial scale of the study), repertoire size, migratory status, whether the song was learnt or not (as we originally retained also studies about vocal non‐learners), territoriality, sexual dichromatism, and information on the habitat of the recording locations. As the methods used by the studies were very heterogeneous, we used a few control variables to test and control for this heterogeneity. Specifically, we categorized response variable type (song or song element sharing) and the subject of comparison (whether song sharing was calculated among individuals or populations/groups). For sample size, we extracted the number of units (groups or individuals) used to calculate the effect size. Unfortunately, data were insufficient for performing meaningful comparisons for the following variables: learnt or not learnt songs (so we excluded studies on vocal non‐learners) and territoriality (categorized as territorial, cooperative breeder, lekking, or flocking based on information from the focal studies).

Because repertoire size was quantified inconsistently across studies, we used broad categories to facilitate interspecific comparisons. During categorization we prioritized repertoire size estimates from the same study or population as the focal effect size, or from previous comparative studies and meta‐analyses (Read and Weary 1992; Sorensen et al. 2016). When no published information was available (for two species), we examined at least 10 recordings from the xeno‐canto database to estimate typical repertoire size (https://xeno‐canto.org/). Repertoire size categories were defined as single (usually one invariable song type), low (3–10 song or element types), middle (11–100), and high (> 100). These categories were based on broad ranges of repertoire size in avian vocal communication literature (Read and Weary 1992; Catchpole and Slater 2008) and were used to facilitate interspecific comparison, to distinguish species with a single or very small repertoire from those with progressively larger repertoires. We used the type of vocal unit corresponding to the response variable in each study; thus, studies quantifying song sharing were classified according to song‐type repertoire size, whereas studies quantifying song‐element sharing were classified according to the repertoire of song elements.

Migratory status (migratory or sedentary) was assigned according to the population from which recordings were obtained; populations showing both behaviors were classified as partially migratory. When migratory status was not reported in the focal study we searched the literature (Paradis et al. 1998; Mullarney et al. 1999; Garamszegi et al. 2003; Møller et al. 2008; Pap et al. 2015; Kennedy et al. 2016; Usui et al. 2017; Valcu et al. 2021). If no published information was available, we searched the following online sources: Birds of the World (https://birdsoftheworld.org/), eBird (https://ebird.org/), and Avibase (https://avibase.bsc‐eoc.org/avibase.jsp).

Regarding sexual dichromatism, species were categorized as dichromatic or monochromatic (the sexes cannot be differentiated by their plumage). When data on sexual dichromatism were not provided in the focal studies, we used the data of previous meta‐analyses (Jeschke and Kokko 2008; Seddon et al. 2013; Beltran et al. 2022), handbooks (Mullarney et al. 1999), or in a few cases, photos made available on the eBird homepage.

We categorized habitat based on Google Maps imagery, assigning sites to natural, rural, or suburban categories following Batáry et al. (2018). The natural category included natural and semi‐natural habitats with minimal human habitation; the rural category contained modified, usually farmland matrices with low density housing; and suburban areas were characterized by residential areas with > 50% vegetation cover (Batáry et al. 2018). None of the study areas could be assigned to the urban category (> 50% artificial, sealed surface). We examined maps at a standardized scale centered on recording locations and compared them with habitat descriptions in the original studies, noting that many older studies lacked precise coordinates. In a few cases, natural/rural/suburban habitat classification was based on the species' typical habitat, as the focal studies did not contain sufficient information on the location and habitats of the study site.

We downloaded the phylogenetic tree of the involved species from birdtree.org. We applied the taxonomy of the AviList checklist (AviList Core Team 2025), so we merged *Troglodytes brunneicollis* with *Troglodytes aedon* . *Acrocephalus orientalis* was not present on the tree, so we placed it next to its closest relative, *Acrocephalus arundinaceus* . In one case we were constrained to use another species from the same genus ( *Arremon torquatus* instead of *Arremon assimilis* , see Figure 2).

**FIGURE 2:**
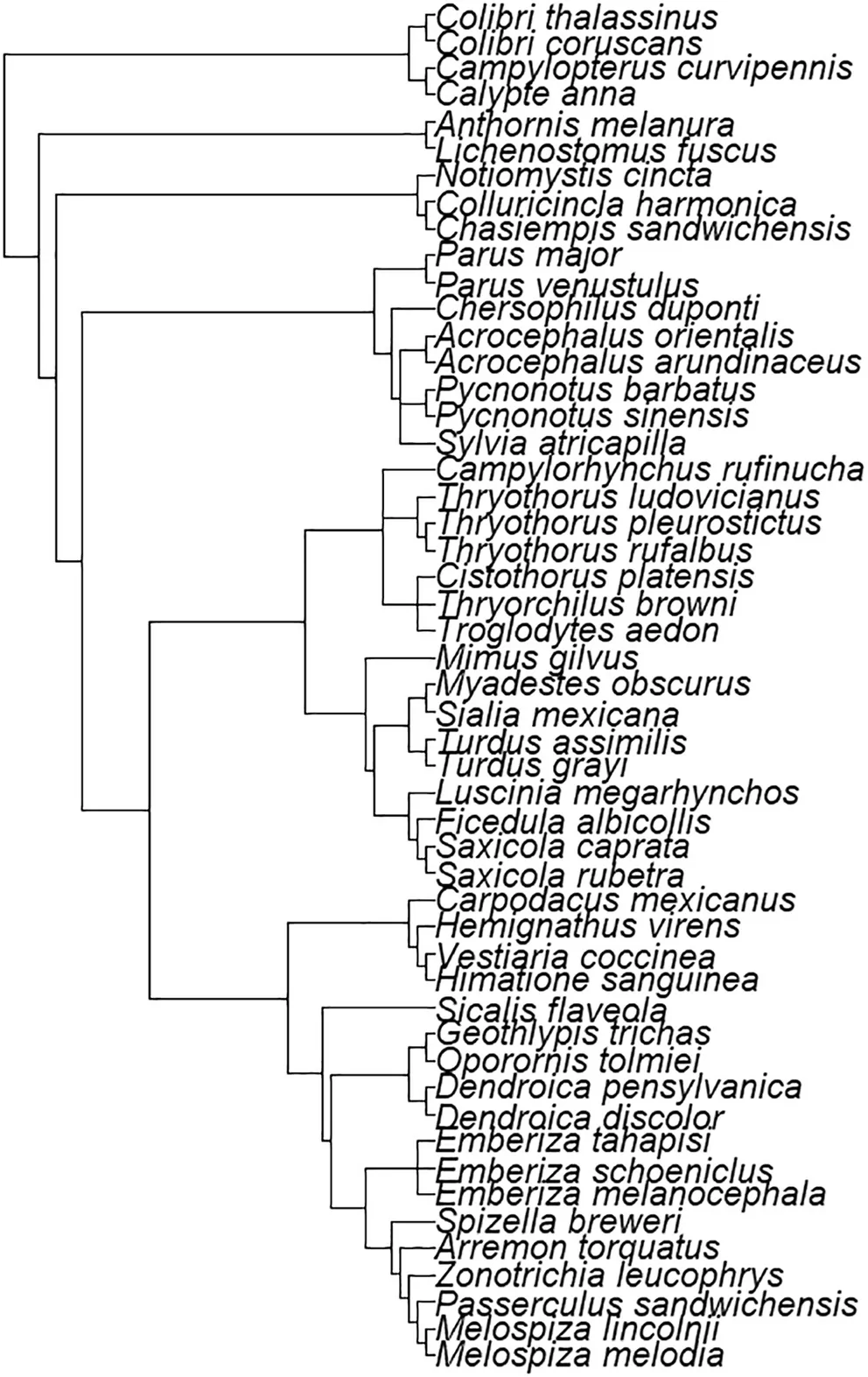
Phylogenetic tree of the bird species included in the meta‐analysis.

### 2.2 Statistical Analyses

We extracted statistical measures suitable for calculating correlation effect sizes from the papers. We used statistics as reported from Mantel‐tests and equivalent methods of matrix comparisons, Spearman and Pearson correlations. The measures that could be converted to correlation effect size were *U* or *z* statistics from the Mann–Whitney *U* test, *T*‐value of Wilcoxon signed rank test, *t*‐value of *t*‐test or Mantel test, *F*‐value of ANOVA comparing a closer and more distant group. The conversions were done based on either https://www.campbellcollaboration.org/calculator/ or https://psychometrica.de/effect_size.html. The signs of the effect sizes were standardized so that positive values indicated decreasing song sharing with increasing geographic distance, whereas negative values indicated greater song divergence among nearby individuals. Correlation effect sizes were converted to Fisher's *Z*‐score. Effect sizes with a sample size of 3 were excluded, as sampling variance could not be estimated for them (*N* = 5), so the final sample size was 97 effect sizes from 58 studies.

First, we built a simple Bayesian phylogenetic meta‐analysis model (Hadfield and Nakagawa 2010; Nakagawa and Santos 2012; Pottier et al. 2024), including no fixed effect and including the species twice (one connected to the phylogenetic tree and one controlling for species‐level effects independent from phylogeny), the independent sampling group ID (to account for potential non‐independence among estimates derived from the same sampling context) and study ID (to account for non‐independence among effect sizes originating from the same study) as random effects (hereafter null model). The variance estimates from the Fisher's *Z*‐score calculation were used as measurement error. From this model we extracted the value of the intercept (the average effect size), phylogenetic signal and heterogeneity (*I* ^2^) (Higgins et al. 2003; Nakagawa and Santos 2012) on the level of species, studies and independent sampling groups.

Next, we tested for publication bias. We included the standard error of the effect sizes as a fixed factor in the above model and found its effect marginally significant (see Section 3), so we repeated this analysis with the variance as a fixed factor (Nakagawa et al. 2022). We also conducted Egger's regression on the marginal residuals (subtracting only the influence of fixed effects from the observations to remove the effect of moderators) of the null model. We also tested the effect of publication year on the effect sizes to check temporal bias in the dataset, including publication year as a fixed factor in the model (Nakagawa et al. 2022). Furthermore, a funnel plot was created depicting the relationship between the standard errors and the marginal residuals (Nakagawa et al. 2022).

As a final step we included the moderators and the methodological control variables into the model as fixed factors. As our sample size was somewhat limited and we wanted to obtain estimates for all levels of the categorical variables, we fitted separate models for each moderator, including one moderator alongside the two control variables. For categorical moderators, the intercept was suppressed so that the model estimated the mean effect size separately for each category. Thus, the posterior estimates for the levels of categorical moderators represent category‐specific mean effect sizes, with credible intervals indicating uncertainty around these estimates. Specifically, these models contained the Fisher Z‐transformed correlation effect size as the response variable, and log‐transformed and scaled maximum distance or migratory status or repertoire size or dichromatism or habitat category as a moderator (see sample sizes on Figure S1), subject of comparison (individual or group) and response category (song or song element) as control variables, along with the same random structure and measurement error as mentioned above. These models did not directly estimate posterior contrasts among categories, so we calculated these by subtracting the posterior distribution of the levels of categorical variables from each other and determining the significance of the differences based on their 95% credible intervals.

We conducted sensitivity analyses to assess the robustness of our results to differences in statistical tests with which the focal relationship was calculated. Although all included relationships reflected associations between song sharing and geographic distance, and so we preferred to include them in the analyses (Pollo et al. 2024), the underlying statistical tests differed substantially. To assess potential bias, we grouped statistical tests by type and included this factor in the null model, and we also repeated the analyses while excluding each test type in turn. These sensitivity analyses indicated that the results were robust (see Text S1, Tables S1–S3).

We built the phylogenetic mixed models using the ‘MCMCglmm’ package (Hadfield 2010). We used priors with an inverse‐Wishart distribution. The models ran for 510,000 iterations, and we set a burn‐in of 10,000 samples, which were discarded at the beginning, and a thinning interval of 500. The trace and distribution of all variables and the autocorrelation between iterations were checked visually. Mixing and convergence were also checked using the Gelman‐Rubin statistics (Gelman and Rubin 1992). We considered a fixed effect estimate significant if the 95% credible interval excluded 0.

All statistical analyses were performed in the R 4.5.0 statistical environment (R Core Team 2023). Fisher's Z scores were calculated with the ‘escalc’ function from the ‘metafor’ package (Viechtbauer 2010). Phylogenetic trees were manipulated with the help of the ‘ape’ package (Paradis and Schliep 2019). The forest plot were drawn using the ‘ggplot’, ‘dplyr’ and ‘ggtext’ packages (Wickham 2016; Wilke and Wiernik 2022; Wickham et al. 2025).

ChatGPT was used to create Figure 4, improve the language and conciseness of the text, and occasionally assist with troubleshooting during the statistical analyses.

## 3 Results

The intercept of the null model (0.260, CI: (0.114, 0.391), see also Table S4) and the phylogenetic signal (*λ* = 0.153, 95% credible interval (CI): (0.003, 0.509)) differed from zero based on the credible intervals. The heterogeneities on the level of species (*I* ^2^ = 5.829, CI: (0.127, 19.242)), on the level of independent sampling groups (*I* ^2^ = 32.458, CI: (9.254, 55.933)), and on the level of studies (*I* ^2^ = 7.796, CI: (0.198, 25.116)) also differed from zero. These three estimates of heterogeneity can be regarded as low, medium, and low, respectively (Higgins et al. 2003).

Tests for publication bias revealed no strong evidence of bias. The relationship between effect size and standard error was marginally non‐significant (estimate: 0.642, CI: (−0.006, 1.303), *p* = 0.062). Further analysis indicated that the variance of effect sizes was significantly related to effect size (0.862, CI: (−0.001, 1.733), *p* = 0.044), although the estimate for the intercept did not change substantially compared to the null model (0.188, CI: (0.032, 0.343), *p* = 0.020). Regarding the Egger's regression, the relationship with precision did not differ from zero (estimate: −0.004, SE: 0.061, *t*‐value = −0.065, *p*‐value = 0.948). Also, there was no evidence that publication year influenced effect sizes (−0.007, CI: (−0.018, 0.003), *p* = 0.212). The funnel plot based on the marginal residuals did not indicate serious bias, either (Figure 3).

**FIGURE 3:**
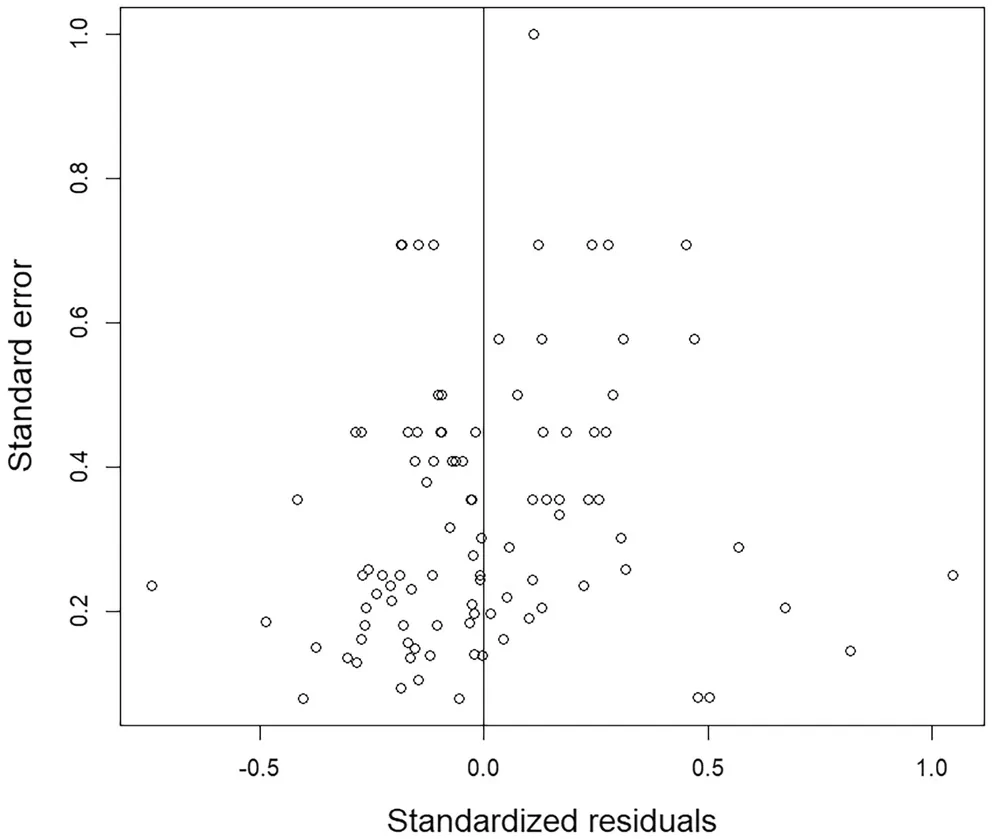
Funnel plot for visualizing potential publication bias showing the standard error of the effect sizes against the marginal residuals.

Multivariate models including the moderators revealed that the effect sizes were significantly positive for all groups, except species with high repertoire size and migrants (Figure 4). Among the control variables, the effect of the response category of song element sharing differed from zero, it was negative (Figure 5). See detailed results of the models in Tables S5–S9. According to the posterior contrasts, effect sizes for partial migrants were stronger positive than for migrants (0.463, CI: (0.131, 0.814)) or residents (0.368, CI: (0.046, 0.684), but note that sample size for partial migrants was only *N* = 6). The effect sizes were also stronger positive for monochromatic than dichromatic species (0.188, CI: (0.008, 0.352)), and in the suburban habitat category than in the natural habitat category (0.264, CI: (0.489, 0.032), see all posterior contrasts in Table S10).

**FIGURE 4:**
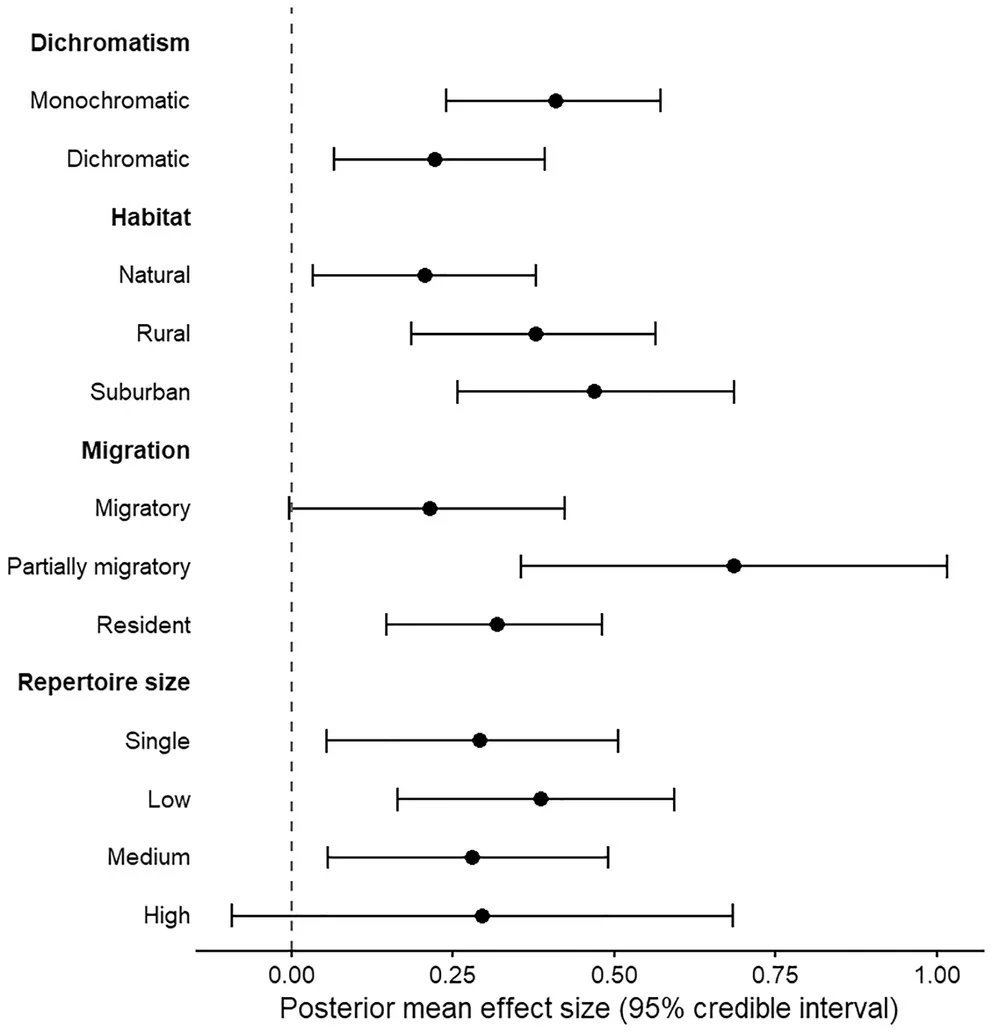
Forest plot showing the results of the meta‐analysis, displaying the posterior mean effect sizes and 95% credible intervals for each level of the categorical moderators (repertoire size, sexual dichromatism, habitat category, migratory status). Note that the signs of the effect sizes were standardized so that positive values indicated decreasing song sharing with increasing geographic distance, whereas negative values indicated greater song divergence among nearby individuals.

**FIGURE 5:**
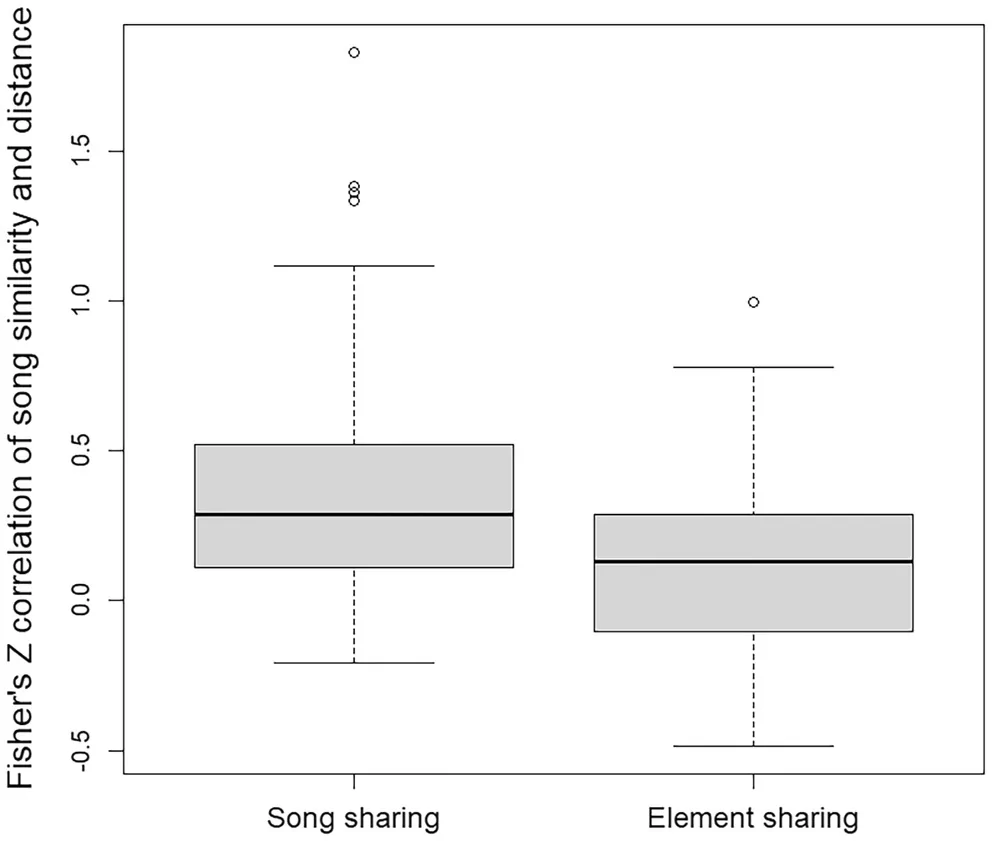
Boxplot showing the relationship between the effect size (the relationship between song distance and geographical distance) and response category (song sharing or song element sharing).

## 4 Discussion

Our meta‐analysis confirmed that song sharing generally decreases with geographic distance. This pattern is likely influenced by multiple factors, with sexual selection and habitat structure appearing to play key roles. The surprising finding that the relationship between song sharing and geographic distance was weaker in dichromatic species suggests that selective pressure for vocal similarity may be weaker in these species. Moreover, a stronger negative relationship was found in suburban habitats, indicating a pressure for higher song sharing.

We found that the investigated bird species generally shared more songs or song elements with closer birds. This pattern likely arises from a combination of evolutionary forces, with sexual selection and habitat effects appearing important based on our results, although the significant (albeit low) heterogeneity among species should be considered when interpreting these findings. Notably, the direction of these relationships is not fully consistent with previous research (Podos and Warren 2007; Wright and Dahlin 2018). Although similar songs in neighborhoods were shown to provide benefits in mate attraction and male–male competition (Baker, McGregor, et al. 1987; Wilson and Vehrencamp 2001; Podos and Warren 2007), we found weaker negative relationship between song sharing and geographic distance in dichromatic species, contrary to the expectation that stronger sexual selection would favor conformity to local song traditions. One possible explanation is that stronger sexual selection may increase the benefits of individually distinctive signals, particularly when males compete for mates or social status (Tibbetts and Dale 2007; Laiolo 2011; Jablonszky et al. 2025), while species in which sexual selection is weaker and have stable neighborhoods can exploit the benefits of high song sharing in male–male contexts. However, this interpretation should be treated cautiously because sexual dichromatism is only an indirect proxy for the intensity of sexual selection. In particular, investment in different signaling modalities may trade off with one another, such that species with stronger visual sexual signals may rely less on song to advertise male quality or identity (Seddon et al. 2013). Thus, our result may reflect differences in the relative importance of visual and acoustic signaling rather than a direct effect of sexual selection on song sharing. More generally, the relationship between sexual selection and song sharing is likely to depend on the social context in which songs are used, including the balance between the benefits of local song conformity and individual distinctiveness and possibly the social structure of the species (e.g., territorial, group‐living). Nevertheless, together with previous work (Beecher et al. 1994; Podos and Warren 2007; Wright and Dahlin 2018), our findings are consistent with an important role of social processes in shaping geographical variation in bird song.

Suburban birds exhibited a stronger negative relationship between song sharing and geographic distance than birds in natural habitats, suggesting that song sharing declines more steeply with distance in these habitats. According to our other prediction, we expected lower levels of song sharing in the suburbs because the fragmentation of the suitable habitat patches and the high level of noise hinders communication (Slabbekoorn and Ripmeester 2008; Laiolo 2010; Marín‐Gómez and MacGregor‐Fors 2021), but this was not supported by our results. One possible explanation for our result is that the heterogeneous mixture of built‐up and vegetated areas characteristic of suburban environments may restrict interactions among individuals or populations and thereby contribute to stronger spatial structuring of song traditions. Habitat fragmentation may affect song sharing also through increased competition among individuals concentrated in remaining suitable habitat patches. However, fragmentation was not quantified in the studies included in our meta‐analysis and can also occur in rural and natural environments. Thus, whether habitat fragmentation contributes to the stronger spatial decline in song sharing observed in suburban habitats remains an open question. Another similar limitation is that we could not investigate the effect of population density, but we did not have reliable data on this for the majority of the study populations. It is also possible that the need to produce songs with good transmission characteristics in the human‐altered environments triggers learning more from successful conspecifics (Slabbekoorn and den Boer‐Visser 2006; Luther and Baptista 2010; Zollinger et al. 2017) and consequently sharing more songs. Yet, to fully explore the extent of the influence of urbanization on the geographic variation in bird song and its proximate causes, we need more studies that quantify the differences in fragmentation, population density, and competition between different habitats.

Although previous studies have suggested that migratory tendencies influence geographic variation in song sharing (Kroodsma et al. 1999, 2002; Nelson et al. 2001; Yoon et al. 2013), we found limited evidence for such an effect. Although the estimated relationship was more strongly negative in partially migratory species, this should be interpreted with caution due to the small sample size and because the resident and migratory species did not differ. The lack of a consistent effect may reflect substantial variation among species in song learning patterns that is independent from migratory status. Species with open‐ended song learning may continue to acquire or modify songs throughout their lives, allowing migratory individuals to learn songs from local neighbors after arriving at their breeding grounds (Ellers and Slabbekoorn 2003; Geberzahn and Hultsch 2004; Beecher and Brenowitz 2005). Thus, migration may not necessarily prevent the formation of local song‐sharing patterns. However, demonstrating open‐ended learning for a species requires time‐consuming experiments, so conclusive data are scarce (but see for example: Todt and Geberzahn 2003; Eriksen et al. 2011; Saldívar et al. 2020). Additionally, species differ in their degree of site fidelity, movement between breeding sites, and turnover of social neighbors, all of which may influence opportunities for song acquisition and sharing. In particular, turnover of neighbors may weaken persistent local song traditions, but may also expose individuals to new tutors and song types. Consequently, migratory status alone may be too coarse a measure to capture the factors that determine opportunities for song sharing.

Although geographic variation in bird song has been studied for decades and used to infer processes such as song learning and population divergence (Irwin 2000; MacDougall‐Shackleton and MacDougall‐Shackleton 2001), the literature remains highly heterogeneous, making broad conclusions difficult. Previous syntheses therefore focused on particular taxonomic groups (Wright and Dahlin 2018) or specific phenomena such as dialects (Podos and Warren 2007). A recent review also highlighted major knowledge gaps, including tropical regions and species and female song (Sebastián‐González and Pérez‐Granados 2025). Although our initial scope was broad, the limited availability of data restricted our analysis to studies of song sharing that conversely allowed us to analyze a more homogeneous dataset. Some heterogeneity nevertheless remained, which we accounted for by controlling for methodological variables (Pollo et al. 2024). Among these, response variable type had a significant effect: effect sizes based on song sharing were stronger negative than those based on song element sharing. This is consistent with songs representing a higher organizational level composed of multiple elements and being more prone to change (Lynch et al. 1989; Burnell 1998; Xing et al. 2013). However, this distinction was not consistently observed in studies examining both song and song element sharing (Sung and Handford 2006; Osiejuk 2011; Linossier et al. 2016). Similarly, Sebastián‐González and Pérez‐Granados (2025) also found an effect of response variable type: geographic differences were stronger for composite acoustic variables than for vocalization type or frequency. We also detected substantial heterogeneity among independent sampling groups containing non‐overlapping sets of individuals, suggesting important population‐level influences on geographic song variation. Future studies should therefore examine song similarity across broader spatial and taxonomic scales while carefully accounting for methodological, species‐specific, and population‐level heterogeneity.

In conclusion, we showed that song sharing is negatively associated with geographic distance; that is, in general, birds share more songs with closer individuals. This pattern was influenced by the strength of sexual selection and the habitat, being stronger negative in monochromatic species and suburban habitats. Thus, in accordance with previous studies, our results also suggest that the geographic variation in bird song is shaped by multiple influencing factors (Podos and Warren 2007; Wright and Dahlin 2018; Sebastián‐González and Pérez‐Granados 2025). However, we could not investigate some factors with potential relevance, such as the presence and type of song learning (Ellers and Slabbekoorn 2003; Beecher and Brenowitz 2005; Sebastián‐González and Pérez‐Granados 2025), genetic drift, or the association with morphological or physiological adaptations that were suggested to contribute to the emergence of dialects as by‐products (Podos and Warren 2007). Future studies should aim to examine more potential mechanisms and evolutionary forces to understand the origin of this wide‐spread phenomenon and its heterogeneity among species.

## Author Contributions

**Mónika Jablonszky:** conceptualization (equal), data curation (lead), formal analysis (lead), investigation (lead), visualization (lead), writing – original draft (lead). **László Zsolt Garamszegi:** conceptualization (equal), formal analysis (supporting), funding acquisition (lead), writing – review and editing (equal).

## Funding

This work was supported by Magyar Tudományos Akadémia, NP2022‐II‐5/2022.

## Conflicts of Interest

The authors declare no conflicts of interest.

## Supporting information


**Text S1:** Testing the effect of the different statistical tests.
**Figure S1:** Distribution of the continuous moderator and sample sizes per category for the categorical moderators.
**Table S1:** The effect of statistical test type (correlation, group comparison, Mantel test and equivalent matrix comparison) on the effect sizes.
**Table S2:** The effect of statistical test type (Mantel test and equivalent matrix comparison, parametric, non‐parametric test) on the effect sizes.
**Table S3:** Average effect size estimates from the null model (intercept) with specific statistical test types excluded from the models.
**Table S4:** Results of the phylogenetic meta‐analysis model with only the intercept (null model). Estimates with 95% credible intervals (CI) and *p*‐values are displayed. The estimate of the intercept with credible intervals excluding zero and *p* value < 0.05 are in bold. Note, that the signs of the effect sizes were standardized so that positive values indicated decreasing song sharing with increasing geographic distance, whereas negative values indicated greater song divergence among nearby individuals.
**Table S5:** Results of the phylogenetic meta‐analysis model with log‐ and z‐transformed maximum distance as moderator. Estimates with 95% credible intervals (CI) and *p*‐values are displayed. Fixed factor estimates with credible intervals excluding zero and *p* value < 0.05 are in bold. The control variables were the subject of comparison (individual or population) and category of the original response variable (song or song element sharing). Note, that the signs of the effect sizes were standardized so that positive values indicated decreasing song sharing with increasing geographic distance, whereas negative values indicated greater song divergence among nearby individuals.
**Table S6:** Results of the phylogenetic meta‐analysis model with migratory status as moderator. Estimates with 95% credible intervals (CI) and *p*‐values are displayed. Fixed factor estimates with credible intervals excluding zero and *p* value < 0.05 are in bold. The 95% credible intervals and pMCMC values indicate whether the estimated category‐specific effects differ from zero; they do not provide direct tests of differences between categories. The control variables were the subject of comparison (individual or population) and category of the original response variable (song or song element sharing). Note, that the signs of the effect sizes were standardized so that positive values indicated decreasing song sharing with increasing geographic distance, whereas negative values indicated greater song divergence among nearby individuals.
**Table S7:** Results of the phylogenetic meta‐analysis model with repertoire size as moderator. Estimates with 95% credible intervals (CI) and *p*‐values are displayed. Fixed factor estimates with credible intervals excluding zero and *p* value < 0.05 are in bold. The 95% credible intervals and pMCMC values indicate whether the estimated category‐specific effects differ from zero; they do not provide direct tests of differences between categories. The control variables were the subject of comparison (individual or population) and category of the original response variable (song or song element sharing). Note, that the signs of the effect sizes were standardized so that positive values indicated decreasing song sharing with increasing geographic distance, whereas negative values indicated greater song divergence among nearby individuals.
**Table S8:** Results of the phylogenetic meta‐analysis model with sexual dichromatism as moderator. Estimates with 95% credible intervals (CI) and *p*‐values are displayed. Fixed factor estimates with credible intervals excluding zero and *p* value < 0.05 are in bold. The 95% credible intervals and pMCMC values indicate whether the estimated category‐specific effects differ from zero; they do not provide direct tests of differences between categories. The control variables were the subject of comparison (individual or population) and category of the original response variable (song or song element sharing). Note, that the signs of the effect sizes were standardized so that positive values indicated decreasing song sharing with increasing geographic distance, whereas negative values indicated greater song divergence among nearby individuals.
**Table S9:** Results of the phylogenetic meta‐analysis model with habitat category as moderator. Estimates with 95% credible intervals (CI) and *p*‐values are displayed. Fixed factor estimates with credible intervals excluding zero and *p* value < 0.05 are in bold. The 95% credible intervals and pMCMC values indicate whether the estimated category‐specific effects differ from zero; they do not provide direct tests of differences between categories. The control variables were the subject of comparison (individual or population) and category of the original response variable (song or song element sharing). Note, that the signs of the effect sizes were standardized so that positive values indicated decreasing song sharing with increasing geographic distance, whereas negative values indicated greater song divergence among nearby individuals.
**Table S10:** Posterior contrasts between levels of categorical moderators. Values represent the posterior mean difference in Fisher's Z‐transformed effect sizes between the indicated categories, with 95% credible intervals in parentheses. Bold contrasts indicate posterior differences whose 95% credible intervals exclude zero. Note, that the signs of the effect sizes were standardized so that positive values indicated decreasing song sharing with increasing geographic distance, whereas negative values indicated greater song divergence among nearby individuals.

## Data Availability

All data and code are provided in Figshare (https://doi.org/10.6084/m9.figshare.31486522). A list of data sources used in the study is provided in the Data sources section.
