# Supplementary material for: Geographic Variation of Song Sharing in Birds—A Meta‐Analysis
Source: Ecol Evol. 2026 Oct 20;16(10):e74566. doi: 10.1002/ece3.74566 (PMC13681252; doi:10.1002/ece3.74566)
Supplement: Supplementary file 1 [file ECE3-16-e74566-s001.docx]

Supplementary material for: Geographic variation of song sharing in birds - a meta-analysis

by Mónika Jablonszky & László Zsolt Garamszegi

Text S1: Testing the effect of the different statistical tests

In order to include all available information into our model we chose to include effect sizes converted to correlation from different statistical methods (Pollo et al. 2024). However, we thoroughly assessed whether the type of the statistical tests could bias our results. Specifically, we included a variable for statistical test type in the null model and investigated its effect. Furthermore, we excluded the specific statistical test types from the data, rerun the null model and examined potential changes in the average effect size. We used two types of categorization for the statistical tests. The first categorization was based on the underlying logic of the test and contained 3 categories: Mantel tests and equivalent matrix comparison, correlation (Spearman, Pearson) and group comparison (Mann-Whitney U test, Wilcoxon signed rank test, t-test and ANOVA). The second categorization consisted of parametric (Pearson correlation, t-test, ANOVA) and non-parametric tests (Spearman correlation, Mann-Whitney U test, Wilcoxon signed rank test) beside Mantel tests. Unfortunately, excluding the most numerous category, Mantel tests (N = 78) was not feasible.

Table S1: The effect of statistical test type (correlation, group comparison, Mantel test and equivalent matrix comparison) on the effect sizes

| Fixed factors | estimate (95% CI), pMCMC value |
| --- | --- |
| Intercept | **0.342** (0.057, 0.637), p = 0.030 |
| Mantel | -0.084 (-0.337, 0.198), p = 0.540 |
| Group comparison | -0.125 (-0.511, 0.199), p = 0.514 |

Table S2: The effect of statistical test type (Mantel test and equivalent matrix comparison, parametric, non-parametric test) on the effect sizes

| Fixed factors | estimate (95% CI), pMCMC value |
| --- | --- |
| Intercept | **0.248** (0.081, 0.414), p = 0.016 |
| Non-parametric | 0.206 (-0.269, 0.293), p = 0.922 |
| Parametric | 0.011 (-0.281, 0.245), p = 0.934 |

Table S3: Average effect size estimates from the null model (intercept) with specific statistical test types excluded from the models

| Excluded method type | estimate (95% CI), pMCMC value | sample size |
| --- | --- | --- |
| Correlation | **0.253** (0.113, 0.396), p < 0.001 | 86 |
| Group comparison | **0.261** (0.071, 0.451), p = 0.028 | 89 |
|  |  |  |
| Parametric | **0.262** (0.114, 0.434), p = 0.010 | 88 |
| Non-parametric | **0.252** (0.084, 0.406), p = 0.018 | 87 |

Supplementary reference

Pollo, P., M. Lagisz, Y. F. Yang, A. Culina, and S. Nakagawa. 2024. Synthesis of sexual selection: a systematic map of meta-analyses with bibliometric analysis. Biological Reviews **99**:2134-2175.

Fig S1: Distribution of the continuous moderator and sample sizes per category for the categorical moderators

Table S4: results of the phylogenetic meta-analysis model with only the intercept (null model). Estimates with 95% credible intervals (CI) and p-values are displayed. The estimate of the intercept with credible intervals excluding zero and p value < 0.05 are in bold. Note, that the signs of the effect sizes were standardized so that positive values indicated decreasing song sharing with increasing geographic distance, whereas negative values indicated greater song divergence among nearby individuals

| Moderators | estimate (95% CI), pMCMC value |
| --- | --- |
| Intercept | **0.260** (0.114, 0.391), p = 0.008 |
| Random factors | Variance (95% CI) |
| Species | 0.007 (0.0002, 0.024) |
| Species (phylogenetic) | 0.016 (0.0002, 0.061) |
| Independent sampling group | 0.040 (0.004, 0.073) |
| Study | 0.010 (0.0003, 0.032) |
| Residual | 0.010 (0.0002, 0.027) |

Table S5: results of the phylogenetic meta-analysis model with log- and z-transformed maximum distance as moderator. Estimates with 95% credible intervals (CI) and p-values are displayed. Fixed factor estimates with credible intervals excluding zero and p value < 0.05 are in bold. The control variables were the subject of comparison (individual or population) and category of the original response variable (song or song element sharing). Note, that the signs of the effect sizes were standardized so that positive values indicated decreasing song sharing with increasing geographic distance, whereas negative values indicated greater song divergence among nearby individuals

| Moderators | estimate (95% CI), pMCMC value |
| --- | --- |
| Intercept | **0.325** (0.168, 0.490), p = 0.002 |
| Maximum distance | 0.057 (-0.039, 0.152), p = 0.264 |
| Comparison (population) | -0.068 (-0.294, 0.170), p = 0.542 |
| Response category (song element) | **-0.171** (-0.311, -0.034), p = 0.026 |
| Random factors | Variance (95% CI) |
| Species | 0.007 (0.0003, 0.026) |
| Species (phylogenetic) | 0.014 (0.0002, 0.060) |
| Independent sampling group | 0.038 (0.008, 0.069) |
| Study | 0.009 (0.0002, 0.029) |
| Residual | 0.008 (0.0002, 0.022) |

Table S6: results of the phylogenetic meta-analysis model with migratory status as moderator. Estimates with 95% credible intervals (CI) and p-values are displayed. Fixed factor estimates with credible intervals excluding zero and p value < 0.05 are in bold. The 95% credible intervals and pMCMC values indicate whether the estimated category-specific effects differ from zero; they do not provide direct tests of differences between categories. The control variables were the subject of comparison (individual or population) and category of the original response variable (song or song element sharing). Note, that the signs of the effect sizes were standardized so that positive values indicated decreasing song sharing with increasing geographic distance, whereas negative values indicated greater song divergence among nearby individuals

| Moderators | estimate (95% CI), pMCMC value |
| --- | --- |
| Migratory status (migrant) | 0.219 (-0.005, 0.441), p = 0.064 |
| Migratory status (partial) | **0.678** (0.310, 1.007), p < 0.001 |
| Migratory status (resident) | **0.324** (0.185, 0.512), p = 0.008 |
| Comparison (population) | -0.029 (-0.231, 0.190), p = 0.762 |
| Response category (song element) | **-0.178** (-0.331, -0.041), p = 0.014 |
| Random factors | Variance (95% CI) |
| Species | 0.010 (0.0002, 0.033) |
| Species (phylogenetic) | 0.013 (0.0002, 0.055) |
| Independent sampling group | 0.021 (0.0003, 0.051) |
| Study | 0.019 (0.0002, 0.055) |
| Residual | 0.008 (0.0002, 0.025) |

Table S7: results of the phylogenetic meta-analysis model with repertoire size as moderator. Estimates with 95% credible intervals (CI) and p-values are displayed. Fixed factor estimates with credible intervals excluding zero and p value < 0.05 are in bold. The 95% credible intervals and pMCMC values indicate whether the estimated category-specific effects differ from zero; they do not provide direct tests of differences between categories. The control variables were the subject of comparison (individual or population) and category of the original response variable (song or song element sharing). Note, that the signs of the effect sizes were standardized so that positive values indicated decreasing song sharing with increasing geographic distance, whereas negative values indicated greater song divergence among nearby individuals

| Moderators | estimate (95% CI), pMCMC value |
| --- | --- |
| Repertoire size (single) | **0.293** (0.072, 0.494), p = 0.022 |
| Repertoire size (low) | **0.381** (0.170, 0.583), p = 0.004 |
| Repertoire size (middle) | **0.278** (0.071, 0.481), p = 0.016 |
| Repertoire size (high) | 0.323 (-0.079, 0.712), p = 0.088 |
| Comparison (population) | -0.009 (-0.212, 0.209), p = 0.974 |
| Response category (song element) | **-0.177** (-0.313, -0.025), p = 0.018 |
| Random factors | Variance (95% CI) |
| Species | 0.007 (0.0002, 0.024) |
| Species (phylogenetic) | 0.018 (0.0002, 0.067) |
| Independent sampling group | 0.038 (0.008, 0.077) |
| Study | 0.010 (0.0003, 0.032) |
| Residual | 0.009 (0.0002, 0.026) |

Table S8: results of the phylogenetic meta-analysis model with sexual dichromatism as moderator. Estimates with 95% credible intervals (CI) and p-values are displayed. Fixed factor estimates with credible intervals excluding zero and p value < 0.05 are in bold. The 95% credible intervals and pMCMC values indicate whether the estimated category-specific effects differ from zero; they do not provide direct tests of differences between categories. The control variables were the subject of comparison (individual or population) and category of the original response variable (song or song element sharing). Note, that the signs of the effect sizes were standardized so that positive values indicated decreasing song sharing with increasing geographic distance, whereas negative values indicated greater song divergence among nearby individuals

| Moderators | estimate (95% CI), pMCMC value |
| --- | --- |
| Dichromatism (no) | **0.408** (0.250, 0.561), p < 0.001 |
| Dichromatism (yes) | **0.222** (0.062, 0.390), p = 0.012 |
| Comparison (population) | -0.042 (-0.234, 0.180), p = 0.694 |
| Response category (song element) | **-0.142** (-0.271, -0.004), p = 0.044 |
| Random factors | Variance (95% CI) |
| Species | 0.006 (0.0003, 0.020) |
| Species (phylogenetic) | 0.008 (0.0002, 0.030) |
| Independent sampling group | 0.036 (0.006, 0.071) |
| Study | 0.008 (0.0002, 0.026) |
| Residual | 0.009 (0.0003, 0.027) |

Table S9: results of the phylogenetic meta-analysis model with habitat category as moderator. Estimates with 95% credible intervals (CI) and p-values are displayed. Fixed factor estimates with credible intervals excluding zero and p value < 0.05 are in bold. The 95% credible intervals and pMCMC values indicate whether the estimated category-specific effects differ from zero; they do not provide direct tests of differences between categories. The control variables were the subject of comparison (individual or population) and category of the original response variable (song or song element sharing). Note, that the signs of the effect sizes were standardized so that positive values indicated decreasing song sharing with increasing geographic distance, whereas negative values indicated greater song divergence among nearby individuals

| Moderators | estimate (95% CI), pMCMC value |
| --- | --- |
| Habitat (natural) | **0.208** (0.039, 0.385), p = 0.028 |
| Habitat (rural) | **0.378** (0.179, 0.587), p = 0.004 |
| Habitat (suburban) | **0.470** (0.268, 0.699), p = 0.002 |
| Comparison (population) | 0.013 (-0.193, 0.218), p = 0.904 |
| Response category (song element) | **-0.213** (-0.375, -0.074), p = 0.002 |
| Random factors | Variance (95% CI) |
| Species | 0.005 (0.0002, 0.016) |
| Species (phylogenetic) | 0.011 (0.0003, 0.041) |
| Independent sampling group | 0.032 (0.001, 0.058) |
| Study | 0.008 (0.0003, 0.024) |
| Residual | 0.009 (0.0002, 0.030) |

Table S10: Posterior contrasts between levels of categorical moderators. Values represent the posterior mean difference in Fisher's Z-transformed effect sizes between the indicated categories, with 95% credible intervals in parentheses. Bold contrasts indicate posterior differences whose 95% credible intervals exclude zero. Note, that the signs of the effect sizes were standardized so that positive values indicated decreasing song sharing with increasing geographic distance, whereas negative values indicated greater song divergence among nearby individuals

| Comparison | Posterior mean (95% credible interval) |
| --- | --- |
| Partial migrant – Migrant | **0.463** (0.131, 0.814) |
| Partial migrant – Resident | **0.368** (0.046, 0.684) |
| Migrant – Resident | −0.104 (−0.285, 0.085) |
| Single – Low repertoire size | −0.093 (−0.290, 0.088) |
| Single – Middle repertoire size | 0.011 (−0.215, 0.231) |
| Single – High repertoire size | −0.007 (−0.410, 0.405) |
| Low – Middle repertoire size | 0.110 (−0.109, 0.316) |
| Low – High repertoire size | 0.087 (−0.299, 0.518) |
| Middle – High repertoire size | −0.015 (−0.393, 0.396) |
| Dichromatic – Monochromatic | **0.188** (0.007, 0.352) |
| Natural – Rural | −0.173 (−0.361, 0.025) |
| Natural – Suburban | **−0.264** (−0.489, −0.032) |
| Rural – Suburban | −0.094 (−0.320, 0.132) |
